# Effect of grape polyphenols on selected inflammatory mediators: A systematic review and meta-analysis randomized clinical trials

**DOI:** 10.17179/excli2020-1011

**Published:** 2020-03-02

**Authors:** Fahimeh Haghighatdoost, Ali Gholami, Mitra Hariri

**Affiliations:** 1 Isfahan Cardiovascular Research Center, Cardiovascular Research Institute, Isfahan University of Medical Sciences, Isfahan, Iran; 2 Noncommunicable Diseases Research Center, Neyshabur University of Medical Sciences, Neyshabur, Iran; 3 Department of Epidemiology & Biostatistics, School of Public Health, Neyshabur University of Medical Sciences, Neyshabur, Iran

**Keywords:** Interleukin-6 (IL-6), tumor necrosis factor-alpha (TNF-a), high-sensitivity C-reactive protein (hs-CRP), grape polyphenols

## Abstract

Grapes contain different polyphenols and might prevent inflammation by reducing Nitric Oxide (NO) inactivation through antioxidative enzymes. The aim of this article was to demonstrate the effects of grape polyphenols on the selected inflammatory mediators, such as interleukin-6 (IL-6), tumor necrosis factor-alpha (TNF-a), and high-sensitivity C-reactive protein (hs-CRP). To find papers assessing the effects of grape polyphenols on inflammatory mediators, electronic data bases, including ISI web of science, PubMed/Medline, SCOPUS, and Google scholar, were searched up to March 2019. Delphi checklist was used for evaluating the qualities of the included articles. The protocol was registered in PROSPERO (No. CRD42019116695). The mean changes in the intervention and control groups were calculated by subtracting the end values from the baselines. Then, the difference between the two changes was measured and utilized as the effect size in meta-analysis. 9 and 8 articles were included in the systematic review and meta-analysis, respectively. Our results indicated that grape polyphenols did not reduce hs-CRP levels, but omission of one article could lead to a significant reduction in hs-CRP (Weight Mean Difference (WMD): −0.54 mg/L, 95 % CI: −1.02, -0.06; P=0.026, I^2^=0.0 %). Regarding IL-6 and TNF-α, no significant changes were observed in the intervention compared to the control group (WMD: 0.04 pg/mL, 95 % CI: −0.02, 0.28; P=0.744, I^2^=0.0 %, WMD: -0.10 pg/mL, 95 % CI: −0.25, 0.05; P=0.183, I^2^=0.0 %, respectively). We found no beneficial effects of grape polyphenols on the selected inflammatory mediators. Still, more studies with higher doses of polyphenols, longer treatment durations, different sources of grape polyphenols, and larger numbers of participants are required.

## Introduction

Chronically increased inflammation reflected by the enhancements of cytokines, endothelial activation markers, and acute-phase proteins is a well-known risk factor for various chronic diseases, such as Coronary Vascular Disease (CVD) (Pearson et al., 2003[38]; Ridker et al., 2004[42]), endothelial dysfunction, metabolic syndrome, and type 2 diabetes (Tsoupras and Lordan, 2018[54]). High sensitive-C-Reactive Protein (hs-CRP), interleukin-6 (IL-6), and tumor necrosis factor-alpha (TNF-α) are the most important inflammatory mediators in relation to health status. IL-6 and TNF-α secreted from lymphocytes and macrophages (Bradley, 2008[5]; Gabay, 2006[14]), as well as liver cells, were stimulated to secrete CRP into blood circulation (Pepys and Hirschfield, 2003[39]) and consequently lead to chronic diseases. 

Evidence suggests that greater food consumptions with higher antioxidant contents can effectively alleviate oxidative stress and inflammation (Haghighatdoost and Hariri, 2019[19]; Salehi-Abargouei et al., 2017[43]; Spormann et al., 2008[50]). Phenolic compounds, such as flavonoids, can slow down or inhibit inflammatory processes via donating electrons or hydrogen (Gulcin, 2012[18]). Epidemiological studies have indicated that the subjects who have consumed higher amounts of flavonoid-rich foods have had lower CVD risks (Geleijnse et al., 2002[15]; Hertog et al., 1993[22]), which might be attributable to the reductions of inflammation and oxidative stress (Cao et al., 1998[7]). 

Polyphenols, which are naturally found in vegetables and fruits, have anti-inflammatory, hypolipidemic, and antioxidant effects (Zern and Fernandez, 2005[58]). Furthermore, in vitro studies have demonstrated that polyphenols can prevent the expressions and secretions of inflammatory mediators (Santangelo et al., 2007[44]). Grapes have different polyphenols, such as flavonols, flavans, anthocyanins, and stilbenes (e.g., resveratrol), and might prevent oxidative stress, LDL oxidation, inflammation, and dyslipidemia (Haghighatdoost and Hariri, 2018[20], 2019[19]; Xia et al., 2010[57]; Zern et al., 2005[59]). Grape polyphenols, especially resveratrol, can reduce inflammation by decreasing Nitric Oxide (NO) inactivation through antioxidative enzymes, including superoxide dismutase, NADPH oxidase, and glutathione peroxidase (Spanier et al., 2009[49]).

There are many Randomized Clinical Trials (RCTs) in human beings for studying the effects of grape polyphenols on inflammatory mediators, but they have yielded conflicting results. A meta-analysis in 2011 with 3 effect sizes indicated that the polyphenols obtained from grape seeds could lower CRP concentrations (Feringa et al., 2011[13]). However, no articles assessing grape polyphenols from other grape derivatives, such as raisin, whole grape extract, and grape juice, were included. The effects of grape polyphenols on other inflammatory mediators were not examined either. Therefore, a meta-analysis is necessary to summarize the overall effects of grape polyphenols on inflammatory mediators. In this systematic review and meta-analysis article, we included all the published RCTs evaluating the effects of grape polyphenols on CRP, IL-6, and TNF-α.

## Materials and Methods

To find papers assessing the effects of grape polyphenols on inflammatory mediators, advanced searches were conducted. Electronic data bases, including ISI web of science, Google scholar, PubMed/Medline, and SCOPUS, were searched up to March 2019 with the following Mesh and non-Mesh key words: 'Interleukin 6', 'IL-6', 'IL6', 'Interleukin-6', 'TNF alpha', 'TNF-alpha', 'Tumor Necrosis Factor-alpha', 'Tumor Necrosis Factor', 'Tumor Necrosis Factor alpha', 'CRP', 'C Reactive Protein', 'Protein, C-Reactive', 'C-Reactive Protein', 'raisin', 'grape', 'grape extract', 'grape juice', 'grape seed extract', 'polyphenol'. We designed an advanced search by using quotation marks, Boolean operators, asterisks, and parentheses. Quotation marks, parentheses, and asterisks were applied to search the exact terms, group search terms, and all the words derived from one keyword, respectively. All the papers found were exported to EndNote software (reference manager software, version X6). To find relevant articles, the titles and abstracts of the exported articles were read by MH and FH separately, while the reference lists of all the relevant reviews and RCTs were hand-searched. PubMed's email alert service was employed to update our searches until May 2019. We solved all discrepancies via a group discussion. This review was performed without any restrictions on the publication time and language.

### Inclusion criteria

We used PICOS framework as the inclusion criteria for this meta-analysis: 1) population: participants aged ≥18, without acute inflammatory diseases, and no gender-based restrictions; 2) intervention: grape polyphenols consumed in any forms of grapes; 3) comparator: placebo; 4) outcomes: serum concentrations of IL-6, TNF-α, and CRP as inflammatory mediators; 5) study design: Randomized Controlled Trials (RCTs).

### Exclusion criteria

Articles with the following criteria were excluded: 1) not reporting polyphenol doses; 2) lacking any data regarding the baseline and end concentrations of the inflammatory biomarkers in the intervention or control groups or any information about changes in the outcomes; 3) having interventions with a duration of less than one day; 4) taking other nutrients beside grape polyphenols only in the intervention group; 5) studying similar populations; 6) not having a control group; 7) taking grape seed oil due to its high vitamin E content (Wen et al., 2016[56]); 8) taking red wine with alcohol instead of dealcoholized red wine; and 9) non-English articles.

### Data extraction

The eligible RCTs for our systematic review and meta-analysis were carefully read and the following data were extracted: country, publication year, first author's name, sample size, sample sizes in the intervention and placebo group, clinical trial design, participant's sex and age, numbers of males and females, participant's health status, dose of grape polyphenols, placebo, treatment duration, mean and Standard Deviation (SD) of serum hs-CRP, and IL-6 and TNF-α levels. All the discrepancies in the extracted data were solved via a group discussion and the MH "Reminder Systems" sent an email to the authors to clarify unclear data.

### Quality assessment

Article quality was scored between 9 (rigorous) and zero (very poor) by using the Delphi checklist (Verhagen et al., 1998[55]). Delphi items included answers to the following questions: I) Did authors perform standard randomization? II) Did they conceal intervention allocation? III) Did they make participants blind? IV) Did they make care providers blind? V) Did they make analyzers blind? VI) Was there any similarity between the control and intervention groups at the beginning? VII) Were there well-defined eligibility criteria? VIII) Did authors present variability of the outcomes? And IX) Did they perform an intention to treat the analysis? 

### Statistical analysis

To calculate the weighted mean difference as the treatment effect, we first extracted the mean changes and their corresponding SDs in serum inflammatory biomarkers both in the control and intervention groups. When no mean changes were reported in the original articles, we calculated them by subtracting the end values from the baselines. In these cases, SD was calculated using the correlation coefficient of 0.6. Then, the difference between the two changes was calculated and used as the effect sizes in the Meta-analysis (Higgins and Green, 2011[23]).

The fixed-effect model was performed to estimate the overall summary effect when there was no evidence of heterogeneity between the studies. However, when I^2^> 25 %, the random-effects model suggested by DerSimonian and Laird was run (DerSimonian and Laird, 1986[10]; Higgins et al., 2003[25]). To assess the statistical heterogeneity, I^2 ^test was applied and the values of greater than 50 % were considered as the substantial heterogeneity (Higgins and Thompson, 2002[24]). A subgroup-analysis was performed to explore the sources of heterogeneity based on health status (healthy vs. unhealthy), sex (male vs. female vs. both), age (< vs. >50 y), duration of study (< vs. ≥median), type of prescribed grape (raisin vs. grape juice vs. grape seed vs. whole grape vs. grape extract), polyphenol dosage (≤ vs. >200 mg/d), and study design (parallel vs. cross-over). The heterogeneity between the subgroups was tested using the fixed-effect model.

A sensitivity analysis was performed to examine the effect of an individual-specific study on the overall effect. Publication bias was evaluated through statistical asymmetry tests (Egger's regression asymmetry test and Begg's adjusted rank correlation test (Egger et al., 2011[11])). P<0.1 indicated a significant publication bias. All the statistical analyses were performed using STATA, version 11.2 (Stata Corp, College Station, TX) and P values of <0.05 indicated the statistical significance.

## Results

**Figure 1:**
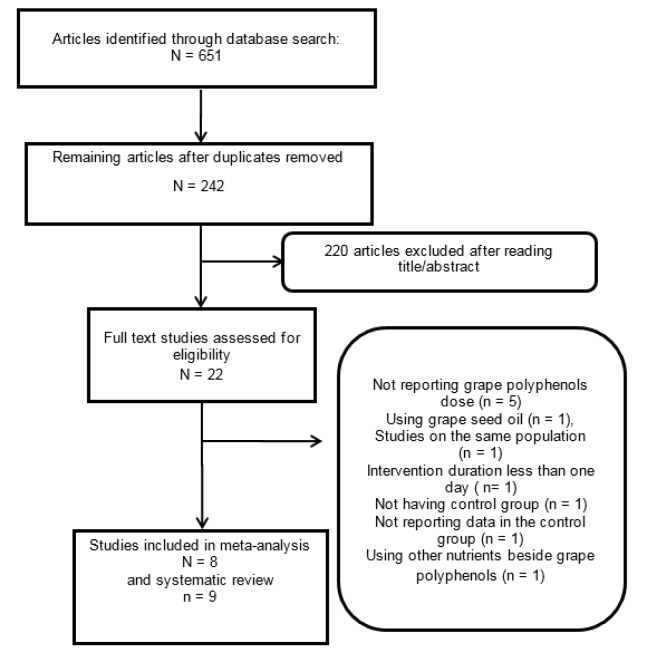
Flow diagram of database searches and study selection

651 articles were retrieved through electronic database searches. After excluding the duplicate articles, 242 articles remained for reading their titles and abstracts. Upon reading the titles and abstracts, 220 articles were excluded and 22 ones were assessed based on the inclusion and exclusion criteria. The full texts of 21 papers were carefully studied and 12 papers were excluded due to the following reasons: not reporting doses of grape polyphenols (n=5), using grape seed oil (n=1), having similar populations (n=1), having intervention durations of less than one day (n=1), not having a control group (n=1), reporting no data for the control group (n=1), using other nutrients beside grape polyphenols (n=1) (Figure 1(Fig. 1)). 

Since the data in the study of Janiques et al. (2014[26]) did not have a normal distribution and we could not calculate the means and SDs, their study was included only in the systematic review, but not in the meta-analysis. Therefore, 9 articles were included in the systematic review (Barona et al., 2012[2]; Castilla et al., 2008[8]; Janiques et al., 2014[26]; Kanellos et al., 2014[28]; Mellen et al., 2010[34]; Tome-Carneiro et al., 2013[52][53]; Zern et al., 2005[59]; and Zunino et al., 2014[60]) and 8 articles in the meta-analysis (Barona et al., 2012[2]; Castilla et al., 2008[8]; Kanellos et al., 2014[28]; Mellen et al., 2010[34]; Tome-Carneiro et al., 2013[52][53]; Zern et al., 2005[59]; and Zunino et al., 2014[60]) (Table 1(Tab. 1); References in Table 1: Barona, 2012[2]; Castilla, 2008[8]; Janiques, 2014[26]; Kanellos, 2014[28]; Mellen, 2010[34]; Tome-Carneiro, 2013[52][53]; Zern, 2005[59]; Zunino, 2014[60]). Grape polyphenols from the whole grape powders or extracts in 6 articles (Barona et al., 2012[2]; Janiques et al., 2014[26]; Tome-Carneiro et al., 2013[52][53]; Zern et al., 2005[59]; and Zunino et al., 2014[60]), red grape juice in one study (Castilla et al., 2008[8]), raisin in one study (Kanellos et al., 2014[28]), and grape seed extract in one study (Mellen et al., 2010[34]) were considered. Our meta-analysis included 436 participants with the treatment durations of 2-48 weeks. The doses of polyphenols ranged from 55 to 640 mg/day. 

In 2 studies conducted by Zern et al. (2005[59]) and Barona et al. (2012[2]), the effects of grape polyphenols were reported separately on 2 different populations; therefore, we enrolled each article as having 2 separate effect sizes. There were 4 study groups (Castilla et al., 2008[8]) in one study on 32 hemodialysis patients. Red grape juice with and without vitamin E was taken in 2 groups, other intervention groups received vitamin E, and the control group received nothing. We considered red grape juice+vitamin E group and vitamin E group as one study and red grape juice and the control group as another; therefore, 2 effect sizes were extracted from that study.

**Figure 2:**
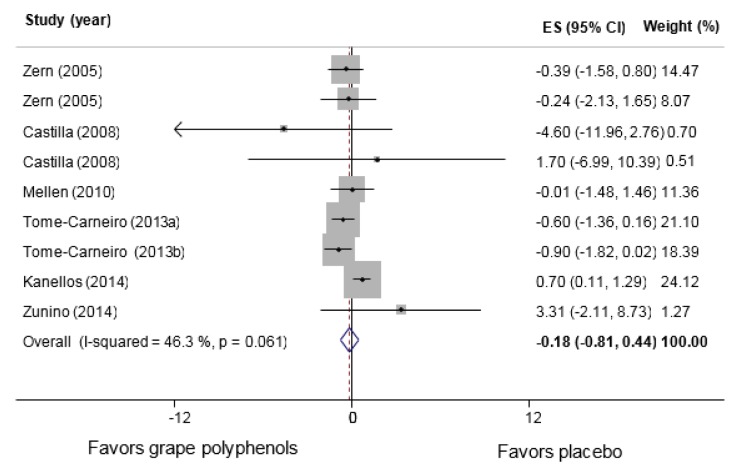
hs-CRP

A total of 7 studies (Castilla et al., 2008[8]; Kanellos et al., 2014[28]; Mellen et al., 2010[34]; Tome-Carneiro et al., 2013[52][53]; Zern et al., 2005[59]; Zunino et al., 2014[60]) with 9 effect sizes and 388 participants (n intervention=198, n control=190) were included in hs-CRP analysis. Our analysis using the fixed-effect model indicated no significant decreases in serum hs-CRP levels (WMD: −0.05 mg/L, 95 % CI: −0.42, 0.32; P= 0.784, I^2^= 46.3 %). Since the between-study heterogeneity was moderate but not significant, we also performed our analysis with the random-effects model, which suggested no significant changes in the intervention compared to the control group (WMD: −0.18 mg/L, 95 % CI: −2.11, 8.73; P=0.569) (Figure 2(Fig. 2); References in Figure 2: Castilla, 2008[8]; Kanellos, 2014[28]; Mellen, 2010[34]; Tome-Carneiro, 2013[52][53]; Zern, 2005[59]; Zunino, 2014[60]). However, the heterogeneity disappeared after omitting the study conducted by Kanellos et al. (2014[28]), which led to a significant reduction in the serum levels of hs-CRP compared with the control group (WMD: −0.54 mg/L, 95 % CI: −1.02, -0.06; P=0.026, I^2^=0.0 %). The results of the subgroup analyses are shown in Table 2(Tab. 2). Significant reductions were found in the studies, which had assessed the effects of grape extract (WMD: −0.72 mg/L, 95 % CI: −1.31, -0.13; P=0.016), and a tendency towards significance was observed in the studies, which had prescribed higher amounts of grape polyphenols (>200 mg/d) (WMD: −0.67 mg/L, 95 % CI: −1.34, 0.01; P=0.053). Significant heterogeneity was found between the subgroups when subgroup analysis was performed based on the intervention type (P=0.019) and polyphenol dose (P=0.033). No significant changes were found in the other subgroups, except for the raisin subgroup included in only one study (Kanellos et al., 2014[28]), which demonstrated a significant increase in the serum levels of hs-CRP following raisin consumption (WMD: 0.70 mg/L, 95 % CI: 0.11, 1.29; P=0.569).

**Figure 3:**
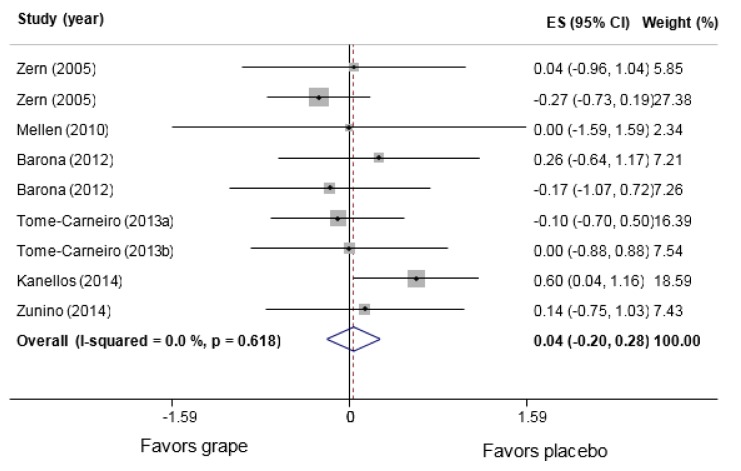
IL-6

**Figure 4:**
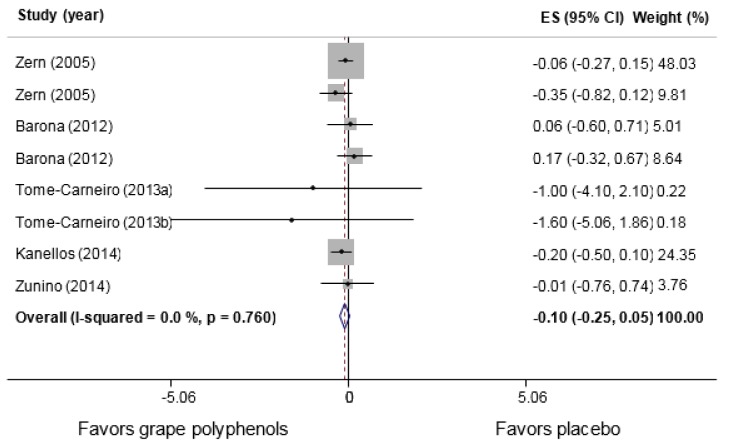
TNF-α

9 comparisons from 7 studies (Barona et al., 2012[2]; Kanellos et al., 2014[28]; Mellen et al., 2010[34]; Tome-Carneiro et al., 2013[52][53]; Zern et al., 2005[59]; Zunino et al., 2014[60]) conducted among 401 participants (n intervention=203, n control=198) assessed the consumption effects of grape polyphenols on serum IL-6 levels. No significant changes were observed in the intervention compared to the control group (WMD: 0.04 pg/mL, 95 % CI: −0.02, 0.28; P= 0.744, I^2^=0.0 %) (Figure 3(Fig. 3); References in Figure 3: Barona, 2012[2]; Kanellos, 2014[28]; Mellen, 2010[34]; Tome-Carneiro, 2013[52][53]; Zern, 2005[59]; Zunino, 2014[60]). The subgroup analyses indicated no changes in the circulatory levels of IL-6 following its consumptions compared with any subgroups in the control group (Table 2(Tab. 2)). Nevertheless, the results obtained in the study of Kanellos et al. (2014[28]) as the only study assessing raisin revealed a significant increase in serum IL-6 levels (WMD: 0.60 pg/mL, 95 % CI: 0.04, 1.16; P=0.036). The between-group heterogeneity was insignificant in all the subgroup analyses. Figure 4(Fig. 4) (References in Figure 4: Barona, 2012[2]; Kanellos, 2014[28]; Tome-Carneiro, 2013[52][53]; Zern, 2005[59]; Zunino, 2014[60]) illustrates the consumption effects of grape polyphenols on serum TNF-α compared with the control group. As displayed, their consumptions are not associated with any significant changes in the serum concentrations of TNF-α (WMD: -0.10 pg/mL, 95 % CI: −0.25, 0.05; P=0.183, I^2^=0.0 %). The subgroup analyses further showed that their consumptions could significantly reduce TNF-α in older adults (WMD: -0.25 pg/mL, 95 % CI: −0.50, -0.01; P=0.045, I^2^=0.0 %), but not in younger individuals (WMD: -0.02 pg/mL, 95 % CI: −0.20, 0.16; P=0.853, I^2^=0.0 %). However, the heterogeneity between the subgroups was not significant (P=0.130).

### Publication bias and sensitivity analysis 

Egger's regression tests demonstrated no evidence of publication bias for any of inflammatory biomarkers (hs-CRP Egger's test: P=0.702; IL-6 Egger's test: P=0.657; TNF-α Egger test: P=0.479). The sensitivity analyses revealed no specific studies with substantial overall effects for IL-6 and TNF-α. Nonetheless, hs-CRP was shown to be significantly reduced with no between-study heterogeneity (WMD: −0.54 mg/L, 95 % CI: −1.02, -0.06; P=0.026, I^2^=0.0 %), except for the study carried out by Kanellos et al. (2014[28]).

## Discussion

According to the electronic database search results, our systematic review and meta-analysis were the first ones, which summarized the effects of grape polyphenols on the selected inflammatory mediators. Our results showed that grape polyphenols could not effectively reduce hs-CRP, IL-6, and TNF-α; however, excluding one article by Kanellos et al. (2014[28]), the serum levels of hs-CRP were found to be significantly lowered. The results of the subgroup analyses revealed that the grape polyphenols obtained from its extract might be more effective in reducing hs-CRP compared with any other forms of grape, while taking its polyphenols at a dose of >200 mg/d showed a tendency towards lower serum concentrations of hs-CRP. Moreover, its consumption by older adults could significantly reduce TNF-α levels, but there were no changes in the serum levels of IL-6 in any of the subgroups.

Grape health-promoting benefits, such as preventing CVD (Shanmuganayagam et al., 2007[46]), protecting against cancer (God et al., 2007[16]), and ameliorating insulin resistance (Martinez-Maqueda et al., 2018[32]), are attributed to its content of polyphenols found in its skin, seed, and stem (Xia et al., 2010[57]). The results of *in vivo* (Terra et al., 2009[51]) and *in vitro* (Chacon et al., 2009[9]) studies have indicated that grape polyphenols have anti-inflammatory effects, which might be related to the immunomodulation pathways, antioxidative pathways (Li et al., 2001[31]), and inhibition of mRNA expression of inflammatory mediators (Terra et al., 2009[51]). It has been well-known that oxidative stress increases inflammation by enhancing the expression of pro-inflammatory mediators and up-regulating the NF-κB pathway (Reuter et al., 2010[41]; Setia and Sanyal, 2012[45]). NF-κB as a transcription factor regulates expressions of numerous genes, which are involved in inflammation (Siomek, 2012[48]). 

The results of our meta-analysis indicated that by excluding the study of Kanellos et al. (2014[28]), grape polyphenols reduced hs-CRP concentrations. Their study was the only one, in which raisin was used as a grape polyphenol source in our meta-analysis. In this research, Corinthian raisin was taken by 48 diabetic patients for 24 weeks. Raisin has high amounts of fructose, which has a low Glycemic Index (GI). Although concerns have been raised that high amounts of fructose, especially among diabetic patients, may have adverse metabolic effects (Sievenpiper et al., 2012[47]), the authors in this study used 36 g/day of raisin, which was higher than its standard serving size for diabetic patients (Esfahani et al., 2014[12]). This might be the reason for increasing inflammatory mediators after 24 weeks of intervention. Furthermore, raisin has a rich source of different polyphenols compared with grape extract or juice (Karadeniz et al., 2000[29]), while the content of each polyphenol in Corinthian raisin depends on its drying process (Panagopoulou and Chiou, 2019[37]). 

According to our subgroup analyses, taking grape polyphenols at a dose of 200 mg/d has a tendency to lower hs-CRP concentration. Polyphenols in foods are mainly found in glycosylated or polymer forms; therefore, 90-95 % of dietary polyphenols cannot be absorbed and thus pass directly to the colon where they are catabolized by gut microbiota and absorbed into the enterocytes (Gu et al., 2004[17]; Monagas et al., 2010[35]). In a dose-response analysis, some investigators suggested that 306.8 mg/day of grape polyphenols provide sufficient amounts of these bioactive agents to reduce cardiovascular risk factors (Blumberg et al., 2015[4]). However, some others suggested that the effective doses of polyphenols to protect against CVDs vary from 200 to 1500 mg/day (Jimenez et al., 2008[27]). 

Our subgroup analysis on the grape source of polyphenols also indicated that its extract significantly reduced hs-CRP concentration. It has been suggested that fiber-bound phenolics have more bioavailability and provide more beneficial metabolites (Maurer et al., 2019[33]); nevertheless, the articles of grape extract supplementations included in our meta-analysis (Tome-Carneiro et al., 2013[52][53]) did not provide any information on the fiber contents of grape peels. Since treatment duration in those studies was 48 weeks and constant consumption might change metabolism or absorption of polyphenols (Novotny et al., 2017[36]), their beneficial effects might be caused by their constant consumptions rather than their sources. Nevertheless, it should be noted that this result was based on only two trials.

In spite of the insignificant effects of grape polyphenols on TNF-α, it showed a decrease among the participants with a mean age of 50 years. TNF-α is a proinflammatory cytokine, which has an important role in the pathogenesis of atherosclerosis. It is an early inflammatory mediator that causes the production of other inflammatory mediators, such as IL-6 and hs-CRP (Biasucci et al., 1996[3]; Harris et al., 1999[21]). Furthermore, TNF-α induces mRNA expression of other cytokines like IL-6 by endothelial cells (Krishnaswamy et al., 1999[30]). Serum concentration of TNF-α is also increased with aging (Bruunsgaard et al., 2000[6]), and thus, its significant reduction in older adults might be due to its higher concentrations in their bodies compared with younger adults. 

In our meta-analysis, grape polyphenols could not reduce IL-6 and our subgroup analysis did not indicate any significant changes in IL-6 levels either. Grape polyphenol dose in all the articles that assessed IL-6 levels was less than 306.8 mg/day, a dose providing a sufficient intake for alleviating cardiovascular risk factors (Blumberg et al., 2015[4]). Therefore, the insignificant effects found in most studies might be due to the low doses of grape polyphenols used in them. 

Our article has several limitations that should be taken into account. 1) All the RCTs included in our meta-analysis had a small sample size, which varied from 11 to 50 participants; therefore, more clinical trials with larger sample sizes are necessary. 2) The majority of the included studies did not contain any information related to the participants' lifestyles, such as physical activity, smoking, and diet. The effects of grape polyphenols on inflammatory mediators might be influenced by different lifestyles as well (Adriouch et al., 2018[1]; Puglisi et al., 2008[40]). 3) Although the doses of polyphenols varied between 55 and 640 mg/day, more articles with higher doses are needed. 4) Most studies did not include any information relevant to dietary fiber contents or grape peels, while fibers can increase the beneficial effects of polyphenols; therefore, providing fiber-related information could be valuable. 5) There was a small number of studies in our subgroup analyses, especially based on polyphenol sources. Since polyphenols in juice, raisin, seed, grape extract, and whole grape are different, more trials on each polyphenol source are necessary to compare their effects on inflammatory mediators. 

Our article has several strengths: 1) It is the first one to measure the effects of grape polyphenols on IL-6, TNF-α, and hs-CRP levels. 2) There were no publication biases for all the selected inflammatory mediators. 3) We had no limitations on the publication times or the languages of the articles. 4) We excluded those using other supplements beside grape polyphenols that might affect the net effects of grape polyphenols on inflammatory biomarkers. 5) There was no heterogeneity in our analysis; and therefore, we reached at firm results.

In conclusion, we found no beneficial effects of grape polyphenols on the selected inflammatory mediators. Given that the doses of higher than 200 mg/day tend to reduce hs-CRP levels, more RCTs with higher doses of polyphenols are required to make a firm decision on the effects of grape polyphenol doses on inflammation. In addition, more RCTs with longer treatment durations, grape polyphenols from different sources, and larger numbers of participants are required. Since 90-95 % of the dietary polyphenols pass directly to the colon to be catabolized by microbiota, future studies should consider dietary prebiotic contents as a confounding factor. 

## Financial support

This meta-analysis and systematic review was financially supported by Neyshabur University of Medical Sciences (Grant number: 98-01-111, Ethical code: IR.NUMS.REC.1398.026).

## Conflict of interest

There is no conflict of interest.

## Acknowledgement

We are extremely grateful to the data collection team at the Neyshabur University of Medical Sciences.

## Figures and Tables

**Table 1:**
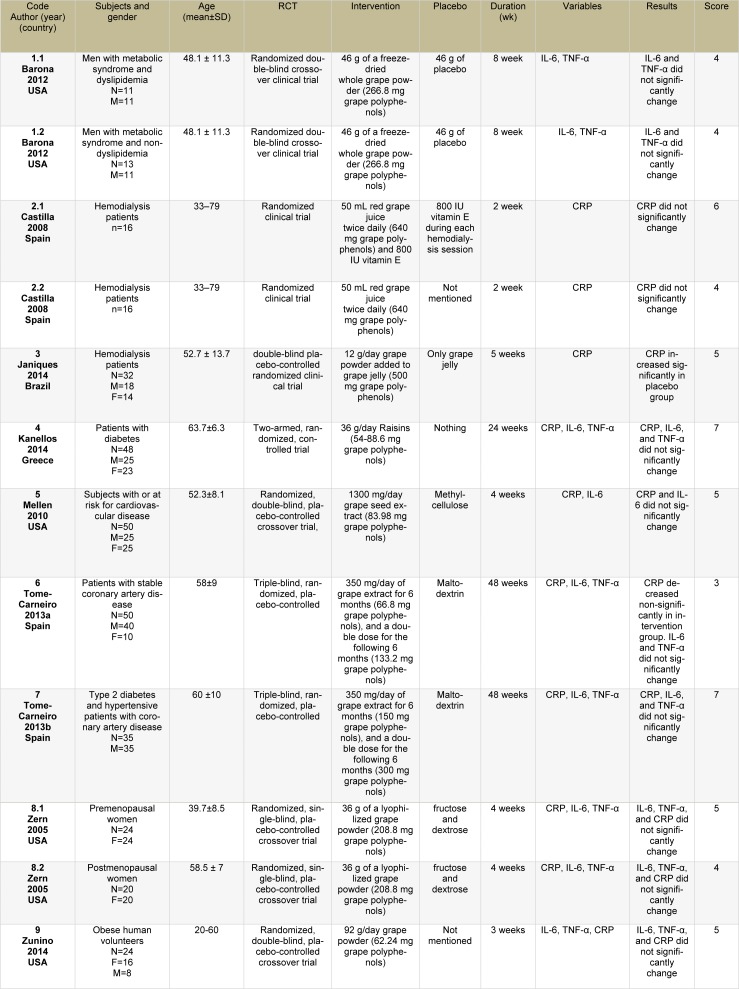
Randomized controlled trial studies included in the systematic review and meta-analysis

**Table 2:**
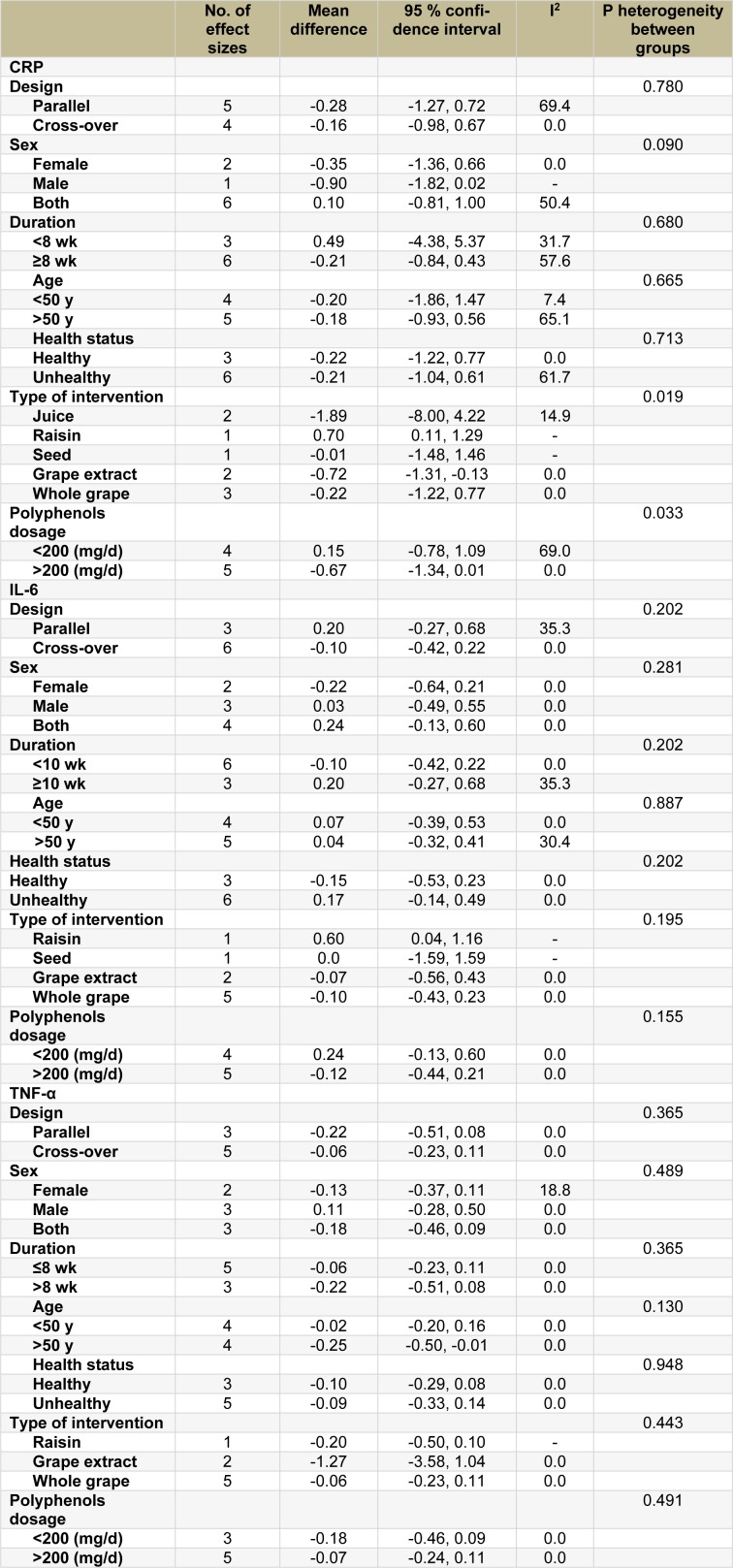
Subgroup analysis for the effect of grape polyphenols on serum inflammatory biomarkers
